# Optimization in computational systems biology

**DOI:** 10.1186/1752-0509-2-47

**Published:** 2008-05-28

**Authors:** Julio R Banga

**Affiliations:** 1 Instituto de Investigaciones Marinas, CSIC (Spanish Council for Scientific Research), C/Eduardo Cabello 6, 36208 Vigo, Spain

## Abstract

Optimization aims to make a system or design as effective or functional as possible. Mathematical optimization methods are widely used in engineering, economics and science. This commentary is focused on applications of mathematical optimization in computational systems biology. Examples are given where optimization methods are used for topics ranging from model building and optimal experimental design to metabolic engineering and synthetic biology. Finally, several perspectives for future research are outlined.

## Background

To optimize means to find the best solution, the best compromise among several conflicting demands subject to predefined requirements (called constraints). Mathematical optimization has been extremely successful as an aid to better decision making in science, engineering and economics.

Optimization and optimality are certainly not new concepts in biology. The structures, movements and behaviors of animals, and their life histories, have been shaped by the optimizing processes of evolution or of learning by trial and error [1,2]. Moreover, optimization theory not only explains current adaptations of biological systems, but also helps to predict new designs that may yet evolve [1,2]. The use of optimization in the close fields of computational biology and bioinformatics has been reviewed recently elsewhere [3,4]. Here, I aim to illustrate the capabilities, opportunities and benefits that mathematical optimization can bring to research in systems biology.

First, I will introduce several basic concepts that can help readers unfamiliar with mathematical optimization. The key elements of mathematical optimization problems are the *decision variables *(those which can be varied during the search of the best solution), the *objective function *(the performance index which quantifies the quality of a solution defined by a set of decision variables, and which can be maximized or minimized), and the *constraints *(requirements that must be met, usually expressed as equalities and inequalities). Decision variables can be continuous (represented by real numbers), resulting in *continuous optimization *problems, or discrete (represented by integer numbers), resulting in integer optimization (also called *combinatorial optimization*) problems. In many instances, there is a mix of continuous and integer decision variables.

As an illustrative example, consider the "diet problem", one of the first modern optimization problems [5], studied in the 1940s: to find the cheapest combination of foods that will satisfy all the daily nutritional requirements of a person. In this classical problem, the *objective function *to minimize is the cost of the food, the *decision variables *are the amounts of each type of food to be purchased (assumed as continuous variables), and the *constraints *are the nutritional needs be satisfied, like total calories, or amounts of vitamins, minerals, etc., in the diet.

The "diet problem" has certain interesting properties: it is a continuous problem where both the objective function (total cost, i.e. sum of the costs of each food purchased) and the constraints are linear with respect to the decision variables, so this problem belongs to the important class of linear programming, or LP (note that due to historical reasons, programming is used here in the sense of planning). These linear constraints define a feasible space (space of decision variables where constraints are satisfied) which is a convex polyhedron, so it is a convex problem. Convex optimization problems [6] are particularly interesting, since they have a unique solution (i.e. they are unimodal) and they can be solved very efficiently and reliably, even for very large number of decisions variables.

**Figure 1:**
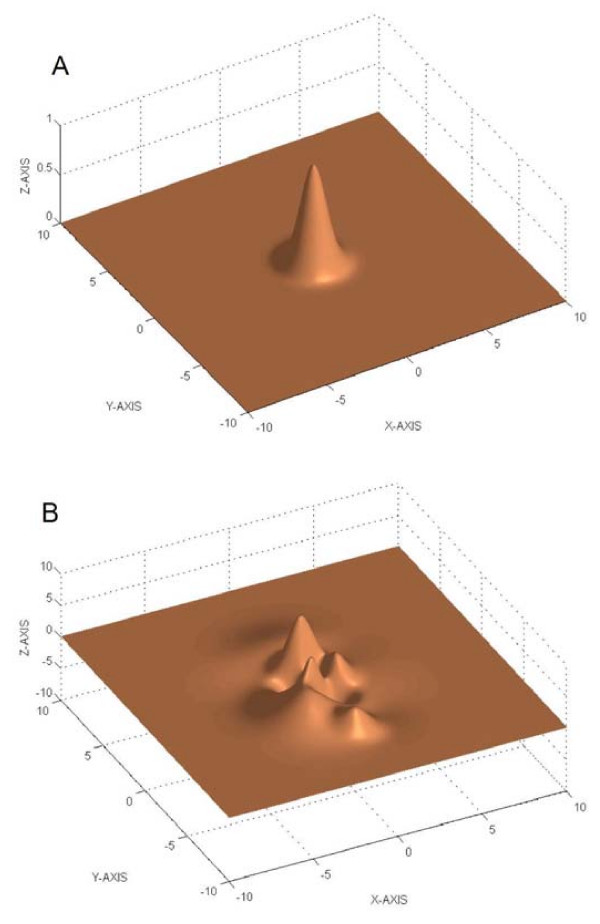
Simple examples (two decision variables, no constraints) of unimodal (1.a) and multimodal (1.b) surfaces, where the z-coordinate of the surface represents the value of the objective function for each pair of decision variables x and y.

Non linear programming (NLP) deals with continuous problems where some of the constraints or the objective function are nonlinear. In contrast to LP, NLP problems are much more difficult to solve. Further, the presence of nonlinearities in the objective and constraints might imply nonconvexity, which results in the potential existence of multiple local solutions (multimodality). Thus, in nonconvex problems one should seek the globally optimal solution among the set of possible local solutions. For the simple case of only two decision variables, one can visualize the objective function of a multimodal problem as a terrain with multiple peaks. Simple examples of unimodal and multimodal surfaces are presented in Figure 1.

The solution of multimodal problems is studied by the subfield of global optimization [7-10]. Many continuous problems and the vast majority of combinatorial optimization problems belong to this class. Most problems in global optimization are very hard to solve exactly in a reasonable computation time. Fortunately, recent developments indicate that convex optimization problems are more prevalent in practice than was previously thought [6]. Thus, it is highly desirable to formulate (or re-formulate) the statement of any optimization problem as a convex one. The book by Boyd and Vandenberghe [6] gives detailed information on how to recognize, formulate, and solve convex optimization problems.

Model-based optimization is a key methodology in engineering, helping in the design, analysis, construction and operation of all kind of devices. Since engineering approaches are playing a significant role in the rapid evolution of systems biology [11-14], it is expected that mathematical optimization methods will contribute in a significant way to advances in systems biology.

In fact, optimization is already playing a key rôle. Examples of applications of optimization in systems biology, classified by the type of optimization problem, are given in Table 1. Below, I highlight several topics where optimization has already made significant contributions.

**Table 1:** Examples of applications of optimization in systems biology, classified by type of optimization problem (note that several types overlap)

**Problem type or application**	**Description**	**Examples with references**
Linear programming (LP)	linear objective and constraints	maximal possible yield of a fermentation [83]; metabolic flux balancing [18,83]; review of flux balance analysis in [30]; use of LP with genome scale models reviewed in [27]; inference of regulatory networks [40,42]
Nonlinear programming (NLP)	some of the constraints or the objective function are nonlinear	applications to metabolic engineering and parameter estimation in pathways [69]; substrate metabolism in cardiomyocytes using ^13^C data [84]; analysis of energy metabolism [85]
Semidefinite programming (SDP)	problems over symmetric positive semidefinite matrix variables with linear cost function and linear constraints	partitioning the parameter space of a model into feasible and infeasible regions [86]
Bilevel optimization (BLO)	objective subject to constraints which arise from solving an inner optimization problem	framework for identifying gene knockout strategies [87]; optimization of metabolic pathways under stability considerations [88]; optimal profiles of genetic alterations in metabolic engineering [89]
Mixed integer linear programming (MILP)	linear problem with both discrete and continuous decision variables	finding all alternate optima in metabolic networks [90,91]; optimal intervention strategies for designing strains with enhanced capabilities [91]; framework for finding biological network topologies [47]; inferring gene regulatory networks [41]
Mixed integer nonlinear programming (MINLP)	nonlinear problem with both discrete and continuous decision variables	analysis and design of metabolic reaction networks and their regulatory architecture [92,93]; inference of regulatory interactions using time-course DNA microarray expression data [45]
Parameter estimation	model calibration minimizing differences between predicted and experimental values	tutorial focused in systems biology [53]; parameter estimation using global and hybrid methods [52,54,55,59,70]; parameter estimation in stochastic models [58]
Dynamic optimization (DO)	Optimization with differential equations as constraints (and possible time-dependent decision variables)	discovery of biological network design strategies [94]; dynamic flux balance analysis [29]; optimal control for modification of self-organized dynamics [95]; optimal experimental design [66]
Mixed-integer dynamic optimization (MIDO)	Optimization with differential equations as constraints and both discrete and continuous decision variables (possibly time-dependent)	computational design of genetic circuits [76]

## Optimization of biochemical reaction networks

Optimization methods have been applied in both metabolic control analysis [15,16] and biochemical systems theory [17]. Further, optimization (and, more in particular, linear programming) has been the engine behind metabolic flux balance analysis, where the optimal flux distributions are calculated using linear optimization, and are used to represent the metabolic phenotype for certain conditions. This flux balance methodology provides a guide to metabolic engineering and a method for bioprocess optimization [18]. Examples of success stories are the *in silico *predictions of *Escherichia coli *metabolic capabilities [19], or the genome-scale reconstruction of the *Saccharomyces cerevisiae *metabolic network [20].

Metabolic engineering exploits an integrated, systems-level approach for optimizing a desired cellular property or phenotype [21]. New optimization-based methods are being developed by using genome-scale metabolic models, which enable identification of gene knockout strategies for obtaining improved phenotypes. However, these problems have a combinatorial nature, so the computational time increases exponentially with the size of the problem for exact methods, so there is a clear need of developing approximate yet faster algorithms [22]. Not surprisingly, optimization will also help in the bioengineering of novel *in vitro *metabolic pathways using synthetic biology, as the key component in rational redesign and directed evolution [23-26].

Coupling constraint-based analysis with optimization has been used to generate a consistent framework for the generation of hypotheses and the testing of functions of microbial cells using genome-scale models [27]. Extensions and modifications of flux balance analysis continue to use optimization methods extensively [28-32].

A particularly interesting question in this context concerns the principles behind the optimal metabolic network operation, *i.e*. "which are the criteria (objective functions) being optimized in these networks?", a question which has been addressed in detail recently [33,34]. Constrained evolutionary optimization has also been used to understand optimal circuit design [35]. Moreover, optimization principles have also been used to explain the complexity and robustness found in biochemical networks [36-38], and much more work in this topic is to be expected in the near future. Related to this, the hypothesis that metabolic systems have evolved optimal strategies as a result of evolutionary pressures has been used in cybernetic models [39], an approach which may offer advantages over traditional methodologies.

## Reverse engineering, modeling and experimental design

Reverse engineering in systems biology aims to reconstruct the biochemical interactions from data sets of a particular biological system. Optimization has been used for inferring important biomolecular networks, such as e.g. transcriptional regulatory networks [40], gene regulatory networks [41-46], signaling pathways [47] and protein interaction networks [48,49].

System identification [50,51] is a methodology widely used in engineering for building mathematical models of dynamical systems based on measured data. Roughly, this involves selected the structure of the model and estimating the parameters of such model from the available experimental data.

The problem of parameter estimation in biochemical pathways, formulated as a nonlinear programming problem subject to the pathway model acting as constraints, has also received great attention [52-59]. Since these problems are frequently multimodal, global optimization methods are needed in order to avoid local solutions. A local solution can be very misleading when calibrating models: it would indicate a bad fit even for a model which could potentially match perfectly a set of experimental data.

Since biological experiments are both expensive and time consuming, it would be ideal if one could plan them in an optimal way, i.e. minimizing their cost while maximizing the amount of information to be extracted from such experiments. This is the purpose of optimal experimental design and optimal identification procedures [60-66], a topic which can make a great impact in the near future, especially in connection with high-throughput techniques.

## Conclusion

Although, as already mentioned, it would be desirable to formulate all the optimization problems as convex ones, in many occasions this is not possible, so we face the solution of global optimization problems, most of which belong to the class of NP-hard problems [67], where obtaining global optima with guarantees will be impossible in many instances. In these situations, approximate techniques like stochastic global optimization can at least locate a near globally optimal solution in a reasonable time, although the cost to pay is that these methods do not offer full guarantees of global optimality. In this context, evolutionary computation methods are a class of stochastic methods which have shown good performance in systems biology applications [55,67-69]. Hybrid methods, combining global and local techniques, have also shown great potential with difficult problems like parameter estimation [54,59,70]. Much more work is needed to further enhance the efficiency and robustness of these approaches in order to make then applicable to large scale models.

Another important issue is the stochasticity that is inherent in biomolecular systems [71,72]. This stochastic nature requires advances in optimization methods, and a number of researches are already providing useful approaches, such as in parameter estimation in stochastic biochemical reactions [58] or in the optimization of stochastic gene network models [73].

As stated in [74], it would be desirable to have computer-aided design tools for biological engineering, similarly to what already happens in many other areas of engineering. Such software would guide the improvement of the behaviour of a biological system *in silico *by optimizing design parameters targeting a selected objective function. The optimization of such synthetic biological systems is in fact receiving increasing attention: optimization algorithms could search for the components (promoters, operators, regulatory proteins, inducers, etc.) and find the best configurations optimizing the dynamic behaviour according to predefined design objectives [75]. A promising example of what can be done is the OptCircuit framework [76], which can be used as an optimization-based design platform to aid in the construction and fine tuning of integrated biological circuits. Other researches are adapting the workflow developed by the electronics industry to the design and assembly of very large scale integrated genetic systems, claiming that the computer assisted design and fabrication of genetic systems will be a reality by 2012 [77].

Moreover, optimization could also be used after the design and construction phases, inside a model predictive control framework [78], to optimally manipulate the resulting biological systems. This is the dream of metabolic engineering [26,79] and synthetic biology [21,25,74]. We are still not there, but the purpose of this paper has been to show that we are getting close. Several issues must be addressed before we reach that goal. First, we need robust and efficient methods for optimization under uncertainty, and for the optimization of stochastic models, that are also able to scale-up, hopefully even at the level of genome-scale models. Second, since neither we nor nature rarely have a single objective, we need multicriteria optimization methods that are better able to cope with the scale and complexity of models from systems biology [80].

Finally, it should be recognized that standard optimization can be sometimes insufficient for gaining deeper insights regarding certain aspects of systems biology, such as in the evolution of biological systems. While evolving towards optimal properties, the environment may change or organisms may even change their own environment, which in turn alters the optimum. In an evolutionary system, continuing development is needed so as to maintain its fitness relative to the systems it is co-evolving with. In other words, everyone has to keep improving in order to survive, which is known as the "Red Queen" effect [81]. Thus, game-theoretic approaches, such as evolutionary game theory [82], may provide a better framework studying the evolution of biochemical systems.

Sutherland [2] claims that, in a context of increasing calls for biology to be predictive, optimization is the only approach biology has for making predictions from first principles. This claim is substantiated by an increasing body of research. We should expect, therefore, even wider use of optimization theory and practice in systems biology.
